# Adapted Chester Step Test Can Have Maximal Response Characteristics for the Assessment of Exercise Capacity in Young Women

**DOI:** 10.3390/healthcare9030308

**Published:** 2021-03-10

**Authors:** Rui Vilarinho, Ana Rita Mendes, Mariana Gomes, Rui Ferreira, Fabíola Costa, Marcela Machado, Márcia Neves, Cátia Caneiras, António Mesquita Montes

**Affiliations:** 1 Center for Rehabilitation Research (CIR), Department of Physiotherapy, Polytechnic Institute of Porto, School of Health, 4200-072 Porto, Portugal; antoniomesquitamontes@gmail.com; 2 Healthcare Department, Nippon Gases Portugal, 2600-242 Lisbon, Portugal; ccaneiras@gmail.com; 3 Private Practice, Polytechnic Institute of Porto, School of Health, 4200-072 Porto, Portugal; anaritamachadomendes.3@gmail.com (A.R.M.); mariana_lg10@hotmail.com (M.G.); ruijsnf@gmail.com (R.F.); fabiola.costa1998@gmail.com (F.C.); marcelapintomachado@hotmail.com (M.M.); marcianeves98@gmail.com (M.N.); 4 Microbiology Research Laboratory on Environmental Health (EnviHealthMicroLab), Faculty of Medicine, Institute of Environmental Health (ISAMB), University of Lisbon, 1649-028 Lisbon, Portugal; 5 Department of Physiotherapy, Santa Maria Health School, 4049-024 Porto, Portugal

**Keywords:** cardiorespiratory fitness, exercise prescription, maximal effort, physical fitness, step test

## Abstract

Chester step test (CST) estimates the exercise capacity through a submaximal response, which can limit its application in the prescription of exercise. This study aimed to assess whether an adaptation of the CST (with a progressive profile) can have maximal response characteristics in young women and compare it to the incremental shuttle walk test (ISWT). Another aim was to determine its within-day test–retest reliability. A cross-sectional study was conducted with 25 women (20.3 ± 1.5 years) who performed the field tests twice on two different days (48 h apart). The maximal effort attainment was assessed by the heart rate (HR), perception of exertion (Borg scale), and blood lactate concentration. For the performance variables, Pearson’s correlation and intraclass correlation coefficient (ICC_2,1_) were used. In the best test, mean values of maximal response were observed in the adapted CST (94.0 ± 6.5% of age-predicted HR_max_, 11.3 ± 4.5 mmol/dl of blood lactate, and 18.4 ± 1.5 of Borg rating). The correlations between the adapted CST and the ISWT were weak to moderate (0.38 ≤ r ≤ 0.55; *p* < 0.05). Fair to good reliability was found for the adapted CST (ICC_2,1_ = 0.48–0.61). The adapted CST showed mean values of maximal response, weak to moderate association with the ISWT, and low within-day test–retest reliability in young women.

## 1. Introduction

The concept of exercise capacity includes the physiological maximal response to an exercise tolerance test with the assessment of cardiorespiratory fitness [1,2]. Step testing is one of the most inexpensive and feasible options to assess exercise capacity [3]. However, most step tests were developed to estimate the exercise capacity through submaximal responses [4]. An adaptation of the Chester step test (CST), a submaximal step test [5,6], to a new and progressive test can be a good option to explore. Furthermore, a progressive test allows the prescription of exercise, especially endurance training, through its maximal work rate achieved [3,7,8]. Taking as example the incremental shuttle walk test (ISWT) [9], this valid field test can reflect the maximal cardiorespiratory response provoked by a cardiopulmonary exercise test (CPET) [10,11,12] allowing the prescription of exercise [3]. Therefore, it is important to confirm in a new and progressive field test whether its peak work rate in performance reflects a maximal physiological response.

The measurement of maximal oxygen uptake (VO_2_max), with the verification of its plateau, is the most important variable to assess the physiological maximal response [3,8,13]. However, when it is not verified, a cluster of other variables can be used to verify the maximal response of a test, such as the respiratory exchange ratio (RER) peak, the achievement of the age-predicted heart rate (HR) maximum, the blood lactate concentration, and the rating perception of exertion [3,14,15]. The assessment of HR, blood lactate, and perception of exertion can be considered a simpler, inexpensive, and not time-consuming cluster of measures to confirm maximal response.

Therefore, the aim of the present study was to assess whether an adaptation of the CST can have maximal response characteristics, through the maximum HR, blood lactate concentration, and rating perception of exertion in young adults, and compare it to the ISWT. Another aim was to determine and compare the within-day test–retest reliability of the adapted CST to the ISWT. The hypothesis is that the adapted CST can be considered a maximum test with a good correlation with the ISWT, but their physiological responses may be different because of their distinct modes of testing (stepping vs. walking). Consequently, different physiological responses can affect the reliability values of the tests.

## 2. Materials and Methods

### 2.1. Study Design, Recruitment, and Study Criteria

A cross-sectional design study was developed involving volunteers’ students (young adults) from a university. The study was advertised at the university, through emails sent to the students’ institutional accounts, so interested participants could contact the research team directly. Only young adults were recruited to reduce the probability of excluding interested participants with disabilities and acute or chronic diseases since their prevalence increase with age [16] and that they can influence the maximal response of an exercise test [3]. Therefore, eligibility criteria were: age between 18 and 30 years, both genders, body mass index between 18.5 and 29.9 kg/m^2^; and physically inactive (who did not perform physical activity for 30 min or more at least three times per week) [3]. Participants with self-report of acute or chronic diseases, smoking habits, pregnant women, flu-like symptoms, or respiratory infection up to four weeks before the study, and currently use of any type of medication were excluded.

This study received ethical approval by a Research Ethics Committee and written informed consent (Declaration of Helsinki) was obtained from all participants.

### 2.2. Measures

The study was performed between January and May of 2019. Each participant performed the adapted CST and the ISWT on different days (48 h apart), randomly. The total time of each collection day for each participant was one hour. Each field test was performed twice (test 1 and test 2) with a rest interval of at least 30 min. Specific instructions were given to the participants: avoid physical activity and any intake of caffeine and alcohol in the 24 h prior to testing, to get at least 8 h of sleep the night before, to eat a light meal and to ingest 500 mL of water in the two hours before the tests. Since the study was conducted during a period of classes, it was not possible to perform the tests at the same period of the day for all participants.

In addition, on the first day, the first procedure was the assessment of the body composition with a segmental bioelectrical impedance analysis (Tanita BC-545 N, Tanita, Amsterdam, The Netherlands), by weight, body mass index (BMI), body fat percentage, and muscle mass measures. For these measures, participants emptied their bladder immediately before the start of the measurements. However, the absence of food or liquid intake for at least 2 h before the measurements was not possible.

### 2.3. The Adapted Chester Step Test and the Incremental Shuttle Walk Test

The ISWT was performed according to the American Thoracic Society recommendations [9] using a 10-m corridor. However, a protocol of 15 levels was used (1500 m) to evaluate adults without disabilities, to prevent the ceiling effect [10]. The test was finished when the participant was not able to maintain the required speed (more than 0.5 m from the cone), when requested by the participant, or for some other reported symptoms of exertion intolerance (dyspnea, leg fatigue, dizziness, vertigo) [9].

In its turn, our step test was adapted from the CST [5,6], using a Max Aerobic step (Mambo, Tisselt, Belgium), with a 0.2 m height. The original test has 5 levels, each of 2 min duration, and the step work rate is set with a metronome, which starts at 15 steps/min and increases by 5 steps/min every 2 min: stage 1 (15 steps/min), stage 2 (20 steps/min), stage 3 (25 steps/min), stage 4 (30 steps/min), and stage 5 (35 steps/min). For the adaptation, it was applied the same initial step work rate as the Chester step test (15 steps/min), but the increment in every 2 min was only by 2 step/min. It was also added the same number of levels as the incremental shuttle walk test (15 levels), with a maximum work rate achievable of 43 step/min. The criteria to stop the test were: not able to maintain the required work rate for 10 s, requested by the participant, or for some other reported symptoms of exertion intolerance (dyspnea, leg fatigue, dizziness, and vertigo) [5,6].

### 2.4. Measurements during the Adapted Chester Step Test Step and the Incremental Shuttle Walk Test

HR and perception of exertion were monitored at rest and during each field test using a cardiofrequencimeter (Polar^®^ FT7, Oy, Finland), and a Borg Rating of Perceived Exertion Scale (range 6–20) [17], respectively. Participants were asked to rate their exertion on the Borg scale combining all sensations and feelings of physical stress and fatigue, disregarding any one factor such as leg pain or shortness of breath [18]. For the analysis of the results, HR and perception of exertion was recorded immediately after the end of each test. Predicted maximum HR (HR_max_) was calculated by the equation HRmax = 220 − age [19], for the calculation of the age-predicted HR_max_ (%). Blood samples were drawn immediately after each test by finger-prick capillary using a lancet and coded yellow test strip with a reagent chemical substance. Blood lactate was analysed by photometry (Accutrend^®^, Roche Diagnostics, Basel, Switzerland) [20].

The performance variables of the adapted CST were the number of steps (primary outcome), maximum work rate reached, and total test time. In its turn, the performance variables of the ISWT were the distance (primary outcome), gait speed reached, and total test time. These performance variables were recorded and presented according to the best test performed by the participants, and also in the first and second attempts (test 1 and test 2).

Trained physiotherapists, with experience in applying these tests, collected the data.

### 2.5. Data, Statistical Analysis

Descriptive and inferential statistical analysis was performed using the IBM’s Statistical Package for the Social Science^®^ software, version 26.0 (IBM Corporation, Chicago, IL, USA), with a significance set at 0.05. All variables were tested for normality using Shapiro–Wilk test. Descriptive data was expressed as mean (standard deviation) for quantitative variables, and absolute and relative frequencies for qualitative variables.

Maximal effort attainment of the adapted CST and the ISWT was analyzed with the mandatory fulfillment of the following three criteria suggested by the literature: (1) attainment of age-predicted maximum heart rate (HR_max_) > 90%; (2) post-exercise blood lactate concentration > 8 mmol/dL; and (3) a Borg Rating of Perceived Exertion >17 [3,14,15].

Paired *t*-test was used to evaluate within-group differences of performance and maximal effort attainment between test 1 and test 2 of each field test. The same statistical test was used to analyze the differences in maximal effort between field tests, in the best test, in test 1 and in test 2.

Pearson’s correlation coefficients were calculated between the adapted CST and the ISWT in their best test. The strength of correlations has been classified according to British Medical Journal guidelines, which regard significant correlation coefficients of 0–0.19 as very weak, 0.2–0.39 as weak, 0.4–0.59 as moderate, 0.6–0.79 as strong, and 0.80–1 as very strong [21].

The sample size was not calculated prior to this study. However, for the association of the adapted CST to the ISWT, we take into consideration the recommendation of David (1938), as cited in Bonnet and Wright (2000), where the use of Pearson’s correlation can be used only if the sample size is equal or superior to 25 [22].

Relative and absolute within-day test–retest reliability was determined for the aCST and ISWT performances. Relative reliability between tests (test 1 and test 2) was determined by intraclass correlation coefficient (ICC) model 2 (two-way random effects), absolute agreement, with a single rater (ICC_2,1_), and with 95% confidence intervals [23]. ICC_2,1_ values were assigned as follows: more than 0.75 = excellent, 0.40–0.75 = fair to good, and less than 0.40 = poor [24]. Absolute reliability was determined by the standard error of measurement (SEM), and the smallest real difference (SRD) [25]. These two last measures also were expressed as percentages (%SEM and %SRD). The SEM was calculated using the equation:

SEM = SD × √(1 − ICC)(1)

where SD is the standard deviation of the performances obtained from all participants, and ICC is the intrarater reliability coefficient. The %SEM was calculated as

%SEM = (SEM/mean) × 100(2)

where “mean” is the mean of the performances obtained in test 1 and test 2. The SRD at the 95% level of confidence was calculated using the equation:

SRD = 1.96 × SEM × √2(3)

The %SRD was calculated as

%SRD = (SRD/mean) × 100(4)

where “mean” is the mean of the performances obtained in test 1 and test 2. An %SRD below 30% has been suggested as an acceptable level of reliability [26].

## 3. Results

Of the 68 volunteers, 27 participants (25 females and 2 males) completed data collection. A total of 41 participants were excluded due to their physical activity levels (*n* = 20), self-report of chronic diseases (*n* = 10), flu-like symptoms in the past four weeks (*n* = 6), and smoking habits (*n* = 5). Due to the predominance of female participants, the 2 males were excluded from the analysis of the results. Participants’ characteristics (*n* = 25) are shown in Table 1. The mean BMI was classified as normal, according to World Health Organization criteria [27].

### 3.1. Performance and Maximal Effort Attainment of adapted Chester Step Test and Incremental Shuttle Walk Test

The mean values of performance and maximal effort of the adapted CST and the ISWT are presented in Table 2. The performance values were significantly higher in test 1 in both tests (*p* < 0.001). All participants performed better in test 1 in the adapted CST, and 23 participants (92%) performed better in test 1 in the ISWT.

Giving the criteria suggested by the literature and according to their mean values, maximal effort attainment was observed in the best test, in test 1, and in test 2 of each field test (Table 2). Significant differences were found in the attainment of age-predicted HR_max_ between test 1 and test 2 in the adapted CST (*p* = 0.001). However, the fulfillment of the three criteria of maximal effort in the adapted CST test was observed in 19 participants (76%) in the best test, 19 participants (76%) in test 1, and 13 participants (52%) in test 2. In the ISWT, the fulfillment of the three criteria of maximal effort was observed in 20 participants (80%) in the best test, 18 participants (72%) in test 1, and 14 participants (56%) in test 2 (Table 2).

Comparing the maximal effort between the two modes of testing in the best test, HR was significantly higher in ISWT (*p* < 0.001), blood lactate concentration was significantly higher in the adapted CST (*p* = 0.03), but no significant differences were observed in Borg rating. In test 1, HR was significantly higher in ISWT (*p* = 0.003), blood lactate concentration was higher in the adapted CST (*p* = 0.04), but no significant differences were observed in Borg rating (*p* = 0.273). On the other hand, in test 2, HR was significantly higher in ISWT (*p* < 0.001), but no significant differences were observed in blood lactate concentration (*p* = 0.312), and in Borg rating (*p* = 0.08) (Table 2).

### 3.2. Correlations between the Tests and Intraclass Correlation Coefficient Values

Table 3 shows the correlations between the best test of the adapted CST (number of steps and level reached) and the ISWT (distance and level reached). These variables had significant positive correlations classified as weak and moderate (0.38 ≤ r ≤ 0.55; *p* < 0.05) (Table 3).

Table 4 presents the relative and absolute reliability results of the aCST and ISWT. According to ICC_2,1_ values, we found fair to good within-day test–retest reliability for the adapted CST, and excellent within-day test–retest reliability for the ISWT. According to the %SRD, the aCST (number of steps) presented a value higher than 30% suggesting an unacceptable level of reliability (Table 4).

## 4. Discussion

One of the main results of the present study showed that the adapted CST had mean values of maximal physiological response in young women. This finding suggests that this adaptation can be used to assess exercise capacity and an alternative to prescribe exercise training through the maximal work rate. To the best of our knowledge, this was the first study to observe the maximal effort attainment in an adapted step test by using the cluster of measures HR, blood lactate, and Borg rating [3,14,15]. However, it is important to mention that some participants did not reach the maximal effort in our adapted CST, as well as in the ISWT. The physical inactivity of the participants may be one of the main factors which contributes to early-onset fatigue and other symptoms (i.e., muscle and joint pain) [28], which leads to the inability to maintain the required work rate, and, consequently, interruption of the tests before obtaining maximal effort.

The differences found between the adapted CST and the ISWT in their physiological responses were expected due to the distinct modes of testing and also by the type of variables used in the cluster of measures, where a central variable (HR) and a peripheral variable (blood lactate) of the cardiopulmonary system were included [3,29,30]. Stepping task, as the stair-ascending, requires a high capacity of the larger thigh muscles, especially the quadriceps, to bear the whole body weight against gravity [31]. The higher blood lactate concentration observed in the adapted CST may reflect this requirement and also suggests greater recruitment of type II fibers (fast-twitch) in this modality, since this type of fibers are predisposed to produce larger quantities of lactate than type I (slow-twitch) fibers [32]. Additionally, a high blood lactate concentration reflects more peripheral activity, as muscle metabolism [33], rather than circulatory responses which are more detected by the raise of cardiac variables, like the HR. Therefore, according to the ISWT response, its higher HR achievement suggests a higher central demand in the cardiopulmonary system [34]. Despite these results, no difference in Borg rating was verified between the tests, which indicates that the tests represented the same perceived general effort by the young women. This finding is explained by the instructions applied in the Borg scale, where participants were asked to rate their exertion combining all sensations and feelings of physical stress and fatigue, disregarding any one factor such as leg pain or shortness of breath [18,35].

One of our interests in the development of this study was also to explore the association between the adapted CST with the ISWT since this last one presents consistent measurement properties and produces a maximal cardiorespiratory response [10,11,12,36]. However, although our results demonstrate that these tests are associated, their correlations were weak to moderate.

Another main result of our study showed that the relative reliability of the adapted CST was fair to good (ICC_2,1_), was lower than the ISWT, and with an unacceptable level of absolute reliability (%SRD), which means that its performance was not reproducible. Furthermore, no learning effect was observed since the mean values of performance were higher in test 1. Even so, we observed mean values of maximal effort already in test 1, which indicates that some young women were able to achieve a maximal response without a familiarization of the test. Therefore, we suggest that one attempt of the adapted CST seems to be enough to assess exercise capacity, but the confirmation of its maximal response is necessary. The length of the test (best test: 23 min) and the high repetitive and intensive stepping of the adapted CST, especially at the end of the test, can explain the lower reliability by the possible inadequate compliance of participants on the second repetition, due to its demanding biomechanical task. The performance of this test can causes high contact forces on joints, especially on the knees, which, consequently, demands the production of greater local muscular exertions when compared to walking/running [31]. Therefore, this greater exertion can cause more local muscle fatigue, especially in sedentary participants, and limit the performance of a second repetition of the adapted CST. According to these findings, we suggest that the rest interval between attempts (30 min) in the adapted CST may not be sufficient, so, more time may be needed to obtain better reliability values.

These low reliability values found in the adapted CST do not reflect the evidence, where higher values for the within-day reliability were observed in other step tests, especially for the 6-min step test in healthy adults (ICC = 0.88–0.90) [37,38]. One explanation is the different types of tests, where the 6-min step test is considered a submaximal test, so the verification of maximal effort attainment, especially higher blood lactate concentration, is less observed. The submaximal characteristic also provides no differences in performance (number of steps) between attempts of the 6-min step test, and, consequently, no learning effect [37,38]. Another observation is that step test performances in disease populations, especially in chronic obstructive pulmonary disease (COPD), presented higher ICC_2,1_ values than our results (ICC > 0.91) [39]. These higher values can be explained by the fact that the step tests applied in COPD are mostly submaximal. On the other hand, in evidence, the reliability of other exercise capacity tests (e.g., 6-min walk test, and the unsupported upper limb exercise test) seems to be higher in disease populations, namely in other chronic lung diseases [40,41,42,43] and cardiovascular diseases [44,45], than in healthy or without disabilities participants [38,46,47]. One of the explanations for these differences is the recruitment process of the samples, as already discussed by Oliveira and colleagues [46], where higher results can be observed in disease populations because these patients are generally recruited from hospitals, which are often more homogeneous in their characteristics than people from the community. However, in our study, we tried to control important confounders in people recruited from the university, involving only young participants (18–30 years old), with BMI classified as normal and overweight, and physically inactive.

As a limitation of our adapted CST, its mean value of total test time (best test: 23.0 ± 2.8 min) was almost twice the time of the ISWT (best test: 12.2 ± 1.0 min). This observation creates a conflict with the general recommendations for the assessment of exercise capacity, which, in general, a test lasting 8–12 min is ideal [14]. This result, adding the suggestion to prolong the rest interval between attempts, can limit the application of the adapted CST in clinical practice by requiring more time. As another limitation, the pre-study sample size was not calculated. However, based on the association between the adapted CST and the ISWT, a post hoc power analysis was conducted. This analysis was based on our achieved sample size (*n* = 25) and the observed effect size (correlation between the primary outcomes of the tests: number of steps on adapted Chester step Test and distance on incremental shuttle walk test, r = 0.40 *p* = 0.04), obtaining a power test of 0.53. Although the power test could have been greater, this current value allowed the verification of significant correlations between the performances of the two tests in their primary outcomes. Even so, the generalizability of our findings has to be confirmed in a larger population, including male participants, and also in a broader range of ages.

Another important suggestion in future studies is to adapt or develop step tests with progressive profiles in disease populations to explore its usefulness to assess exercise capacity and its applicability in exercise training in rehabilitation programs. The assessment of inter-rater reliability should also be addressed in future studies.

## 5. Conclusions

The adapted CST showed mean values of maximal response, has weak to moderate association with the ISWT, and low within-day test–retest reliability in young women. Therefore, a careful application of this test in clinical practice is necessary for the assessment of exercise capacity. Furthermore, it requires more time for its performance than ISWT. Future studies are important with both genders and people with diseases to explore its usefulness.

## Figures and Tables

**Table 1:** General characteristics of participants (*n* = 25).

Characteristics	Young Women Mean ± SD
Age (years)	20.3 ± 1.5
Body mass (kg)	61.7 ± 9.7
Height (m)	1.7 ± 0.1
BMI (kg/m^2^)	22.4 ± 2.6
Total fat mass (%)	25.4 ± 6.2
Lean mass (kg)	43.3 ± 3.9

Data are expressed as mean ± standard deviation. BMI, body mass index.

**Table 2:** Performance and maximal effort attainment results of the adapted Chester step test and incremental shuttle walk test.

Adapted Chester Step Test	Best Test Mean ± SD	Test 1 Mean ± SD	Test 2 Mean ± SD	*p* Value between Test 1 and Test 2
**Performance**				
Number of steps Total test time test (minutes)	602.8 ± 103.3	602.8 ± 103.3	523.5 ± 87.9	<0.001
	23.0 ± 2.8	23.0 ± 2.8	21.2 ± 2.5	<0.001
Level reached	11.2 ± 1.4	11.2 ± 1.4	10.3 ± 1.3	<0.001
**Maximal effort variables**				
HR_max_ (% predicted)	94.0 ± 6.5	94.0 ± 6.5	91.1 ± 6.0	0.001
HR_max_ (bpm)	187.7 ± 13.0	187.7 ± 13.0	182.0 ± 12.3	0.001
Blood lactate (mmol/dl)	11.3 ± 4.5	11.3 ± 4.5	10.0 ± 4.5	0.236
Borg rating	18.4 ± 1.5	18.4 ± 1.5	17.9 ± 1.9	0.103
**Incremental Shuttle Walk Test**	**Best test** **Mean ± SD**	**Test 1** **Mean ± SD**	**Test 2** **Mean ± SD**	***p*** **Value** **between Test 1 and Test 2**
**Performance**				
Distance (m)	1066.2 ± 149.9	1059.9 ± 137.1	1011.0 ± 136.5	<0.001
Total test time test (minutes)	12.2 ± 1.0	12.1 ± 1.0	11.9 ± 0.9	<0.001
Level reached	11.8 ± 1.0	11.8 ± 1.1	11.6 ± 0.9	0.06
**Maximal effort variables**				
HR_max_ (% predicted)	97.6 ± 3.6 *	97.4 ± 3.8 *	96.6 ± 3.3 *	0.06
HR_max_ (bpm)	194.9 ± 7.2 *	194.4 ± 7.6 *	193.0 ± 6.7 *	0.06
Blood lactate (mmol/dl)	9.0 ± 3.0 *	8.9 ± 3.1 *	8.9 ± 3.5	0.898
Borg rating	18.0 ± 1.4	18.0 ± 1.5	17.2 ± 1.9	0.08

Data are expressed as mean ± standard deviation. HR_max_: maximum heart rate. * *p* < 0,05 for comparisons between the adapted Chester step test and incremental shuttle walk test in the best test, in test 1, and in test 2.

**Table 3:** Correlations between the best performance of the adapted Chester step test and incremental shuttle walk test.

Variables	r	*p* Value
aCST number of steps and ISWT distance	0.40	0.04
aCST level reached and ISWT distance	0.55	0.005
aCST number of steps and ISWT level	0.38	0.04
aCST level reached and ISWT level	0.51	0.01

aCST, adapted Chester step test; ISWT, incremental shuttle walk test.

**Table 4:** Results of reliability measures of the adapted Chester step test and incremental shuttle walk test.

Variables	ICC_2,1_ (95% CI)	SEM	SEM (%)	SRD	SRD (%)
**Adapted Chester Step Test**					
Number of steps	0.48 (0.03 to 0.76) *	72.3	12.8	200.4	35.6
Total test time (min)	0.61 (0.04 to 0.85) *	1.7	7.7	4.7	21.3
**Incremental Shuttle Walk Test**					
Distance (m)	0.90 (0.27 to 0.97) *	42.7	4.1	118.4	11.4
Total test time (min)	0.87 (0.50 to 0.96) *	0.33	2.8	0.91	7.6

ICC, intraclass correlation coefficient; SEM, standard error of measurement; SRD, smallest real difference. * *p* < 0.001.

## Data Availability

The data presented in this study are available on request from the corresponding author.
